# The metabolic screening today

**DOI:** 10.1186/1824-7288-40-S1-A63

**Published:** 2014-08-11

**Authors:** Giovanni Sorge

**Affiliations:** 1 Dipartimento di Scienze Mediche e Pediatriche, Unità Operativa Complessa di Clinica Pediatrica, Centro di Riferimento Regionale per la prevenzione, la diagnosi e la cura delle malattie metaboliche congenite dell'infanzia, Università di Catania, Italy

## 

Newborn screening is the process of testing newborn babies for inborn errors of metabolism that include disorders of amino acids, organic acids, fatty acids and urea cycle disorders.

Affected baby are usually normal at birth. Symptoms develop after a latent period ranging from few hours to many years. Many of these disease are potentially fatal conditions and some are extremely rare, but the delay or the lack of diagnosis and treatment can lead to developmental disability, mental retardation and premature death [1,2]

A significant percentage of these conditions (approximately 30-40%) is susceptible to dietary therapy, while in the remaining cases have been proposed various treatment strategies such as enzyme replacement therapy (ERT), vitamin supplementation, organ transplants, in addition to the use of new drugs with specific action.

The neonatal screening by tandem mass spectrometry (expanded newborn screening) allows the early detection of about 50 of these congenital disorders.

The advantages of this method consist in the speed of execution (1-2 minutes for each analysis), the ability to examine numerous analytes in a single spot of blood, the ability to recognize variants "mild" of numerous diseases and then to have updated epidemiological data, a favourable cost effectiveness ratio.

Among the disadvantages we must mention the false positive and false negatives. The first become negligible if the sampling is performed correctly and if the cut-off values of various analytes are re-evaluated periodically, the latter can be attributed to technical errors (sampling time, cut-off or laboratory errors) or to the fact that some of these diseases can not yet recognized by the tandem mass spectrometry [3]

Technological and therapeutic advances during the past 5-10 years have made possible a great expansion of neonatal screening, however, views about which disorder should be included ranging from the five disorders screened in UK to more than 50 recommended in USA [4].

In Sicily, a Regional project, has allowed us to begin the expanded newborn screening, dividing the Region into two areas, east and west, the first assigned to the "Centre for the prevention, diagnosis and treatment of congenital metabolic diseases" of Catania and, the other, to the "Screening Centre" of Palermo. The project includes screening for 30 diseases.

There is a large debate in the scientific literature on the opportunity to introduce neonatal screening for other congenital diseases such as, for example, the lysosomal storage diseases [4-7]
